# Ethanol, Neurodevelopment, Infant and Child Health (ENRICH) prospective cohort: Study design considerations

**DOI:** 10.12715/apr.2015.2.10

**Published:** 2015-04-28

**Authors:** Ludmila N. Bakhireva, Jean R. Lowe, Hilda L. Gutierrez, Julia M. Stephen

**Affiliations:** 1 Department of Pharmacy Practice and Administrative Sciences, University of New Mexico College of Pharmacy, Albuquerque, NM, USA; 2 Department of Family and Community Medicine, University of New Mexico, Albuquerque, NM, USA; 3 Department of Pediatrics, University of New Mexico, Albuquerque, NM, USA; 4 The Mind Research Network and Lovelace Biomedical and Environmental Research Institute, Albuquerque, NM, USA; 5 Department of Neurosciences, University of New Mexico, Albuquerque, NM, USA

## Abstract

**Background:**

While intervention is the leading factor in reducing long-term disabilities in children with fetal alcohol spectrum disorder (FASD), early identification of children affected by prenatal alcohol exposure (PAE) remains challenging. Deficits in higher-order cognitive domains (e.g. executive function) might be more specific to FASD than global neurodevelopmental tests, yet these functions are not developed in very young children. Measures of early sensorimotor development may provide early indications of atypical brain development during the first two years of life.

**Methods:**

This paper describes the novel methodology of the Ethanol, Neurodevelopment, Infant and Child Health (ENRICH) prospective cohort study of 120 maternal-infant pairs with a goal to identify early indices of functional brain impairment associated with PAE. The cohort is established by recruiting women early in pregnancy and classifying them into one of three study groups: patients on opioid-maintenance therapy who consume alcohol during pregnancy (Group 1), patients on opioid-maintenance therapy who abstain from alcohol during pregnancy (Group 2), and healthy controls (Group 3). After the initial prenatal assessment (Visit 1), patients are followed to Visit 2 occurring at delivery, and two comprehensive assessments of children at six (Visit 3) and 20 months (Visit 4) of age. ENRICH recruitment started in November 2013 and 87 women were recruited during the first year. During Year 1, the biospecimen (maternal whole blood, serum, urine, dry blood spots of a newborn) collection rate was 100% at Visit 1, and 97.6% for those who completed Visit 2.

**Discussion:**

The tiered screening approach, evaluation of confounders, neurocognitive and magneto-/electro-encephalography (MEG/EEG) outcomes, and ethical considerations are discussed.

## Introduction

Recent data indicate that as many as 10.7% of pregnant women consume alcohol during pregnancy [1]. The prevalence of fetal alcohol syndrome (FAS) ranges from 0.5–2.0/1000 live births in the general population, to 9.8/1000 live births in high-risk groups [2]. It is well established that children with the facial dysmorphia, characteristic of FAS, have cognitive and behavioral deficits [3]. However, many more children with a history of prenatal alcohol exposure (PAE) also have impaired cognitive processing, even in the absence of facial dysmorphia [4]. The prevalence of this broader phenotype, termed fetal alcohol spectrum disorder (FASD), is at least ten times greater than FAS and might affect up to 4.8% of school-age children [2, 5]. This prevalence underscores the significant need for both earlier and more reliable identification of children with FASD to provide better long-term outcomes [3, 6].

However, in the absence of the characteristic facial dysmorphia, there are currently no reliable biobehavioral markers to identify young children with FASD, which often delays intervention until behavioral deficits become apparent in school-aged children. Streissguth and colleagues (2004) found that children without facial anomalies fared worse in life outcomes than those with dysmorphia due, in part, to a lack of intervention [7]. Finally, early diagnosis and intervention are the leading factors in reducing long-term disabilities in children with FASD [7–11]. Therefore, identifying early indices of atypical brain development in children with known PAE is a critical first step for improving long-term outcomes.

Prior research indicates that global developmental tests, such as the Bayley Scales of Infant Development (BSID), are not sensitive enough to accurately identify early impairment in young children [12]. Children affected by PAE have been found to exhibit slower cognitive processing speed and reaction time, and poorer performance on measures of attention, working memory, and fine motor tasks [13–15]. Early difficulties in self-regulation and working memory are also recognized as part of the behavioral phenotype for children with FASD, as described at the recent Alcohol-Related Neurodevelopmental Disorder (ARND) Consensus Conference [16], and are strongly related to later school failure [17]. Neurodevelopmental tests focusing on these domains, which are now being developed for testing children as early as 18 months to two years of age, may provide a more specific measure of neurocognitive abnormalities associated with higher-order cognitive functions [18].

On the other hand, sensorimotor functions develop earlier than higher cognitive abilities such as executive function or self-regulation [19, 20]. Thus, measures of early sensorimotor development may provide early indications of atypical brain development in very young children. Previous studies have indicated that children with PAE experience altered sensory development [21–23]. For example, in our study of preschool-aged children the auditory evoked response measured with magnetoencephalography (MEG) revealed a delay in auditory processing in the children with FASD relative to healthy control participants [22]. Previous electroencephalography (EEG) studies have identified similar delays in sensory processing in infants known to have been prenatally exposed to alcohol. Therefore, MEG and EEG, as noninvasive measures of neurophysiological responses, provide a means to identify early indicators of altered brain development as a result of PAE. Thus, a combination of MEG/EEG indices with specific neurobehavioral tasks might be a more sensitive measure of functional brain deficits at a young age than either modality alone.

We believe that significant new advances in FASD diagnosis and interventions require a focus on prospective studies of children earlier in development, and an establishment of novel analytical methods for detecting and assessing functional brain damage associated with PAE. This paper describes the novel methodology of a prospective cohort study of pregnant women and their children, followed from birth to 20 months of age, to identify early indices of functional brain damage associated with PAE.

## Methods

### Overview of the study design and study population

The study utilizes a prospective cohort design, which involves recruitment of pregnant women and follow-up of the children born to cohort participants through 20 months of age. The cohort involves four visits: 1) a baseline prenatal visit; 2) assessment during the hospital stay after labor/delivery; 3) six-month assessment of the child; and 4) 20-month assessment of the child. Participants are recruited into three study groups. The alcohol consumption group (Group 1) is comprised of pregnant women on opioid-maintenance therapy (OMT) who are screened and enrolled based on their self-reported alcohol use in the periconceptional period and during pregnancy, and on results from a panel of alcohol biomarkers as described in further detail below. To account for the potential confounding effect of socio-economic status (SES) and postnatal environment, two control groups are being recruited: a) study Group 2 includes opiate-dependent women on OMT who abstained from alcohol use since their last menstrual period (LMP); and b) study Group 3 includes pregnant women who abstained from alcohol use since their LMP, are lifetime non-users of illicit drugs, and lifetime non-smokers. Study Groups 1 and 2 are being recruited from the University of New Mexico (UNM) Milagro clinic, which supports pregnant women with a history of substance/alcohol abuse. Participants for study Group 3 (unexposed controls) are being recruited from the General Obstetrics clinics at UNM. The two control groups will allow for testing the effects of PAE versus other drugs of abuse, and Group 2 will provide the best control for SES.

The following inclusion criteria will apply to patients in all three study groups: participants must 1) be at least 18 years old; 2) have a singleton pregnancy confirmed by ultrasound; 3) be residing and planning to stay in the Albuquerque metropolitan area over the next two years; 4) have the ability to give informed consent in English; 5) have no cocaine, crack-cocaine, or methamphetamine use during the periconceptional period and after LMP (lifetime abstainers from all drug classes for Group 3); and 6) have no fetal diagnosis of a major structural anomaly. Healthy controls (Group 3) are also required to be lifetime non-users of illicit drugs and lifetime non-smokers.

Recruited pregnant women are followed throughout pregnancy. Participants are identified at the time of admission for labor and delivery by communication nursing orders placed in their electronic medical records (EMR). Collection of study-related specimens is tied to the collection of clinical laboratory draws at admission. The second interview occurs during the hospital stay after labor and delivery. In each of the three study groups, children born to cohort participants are followed-up at six and 20 months for neurodevelopmental and neuroimaging assessment, as described below.

### Screening and group allocation

**Figure 1:**
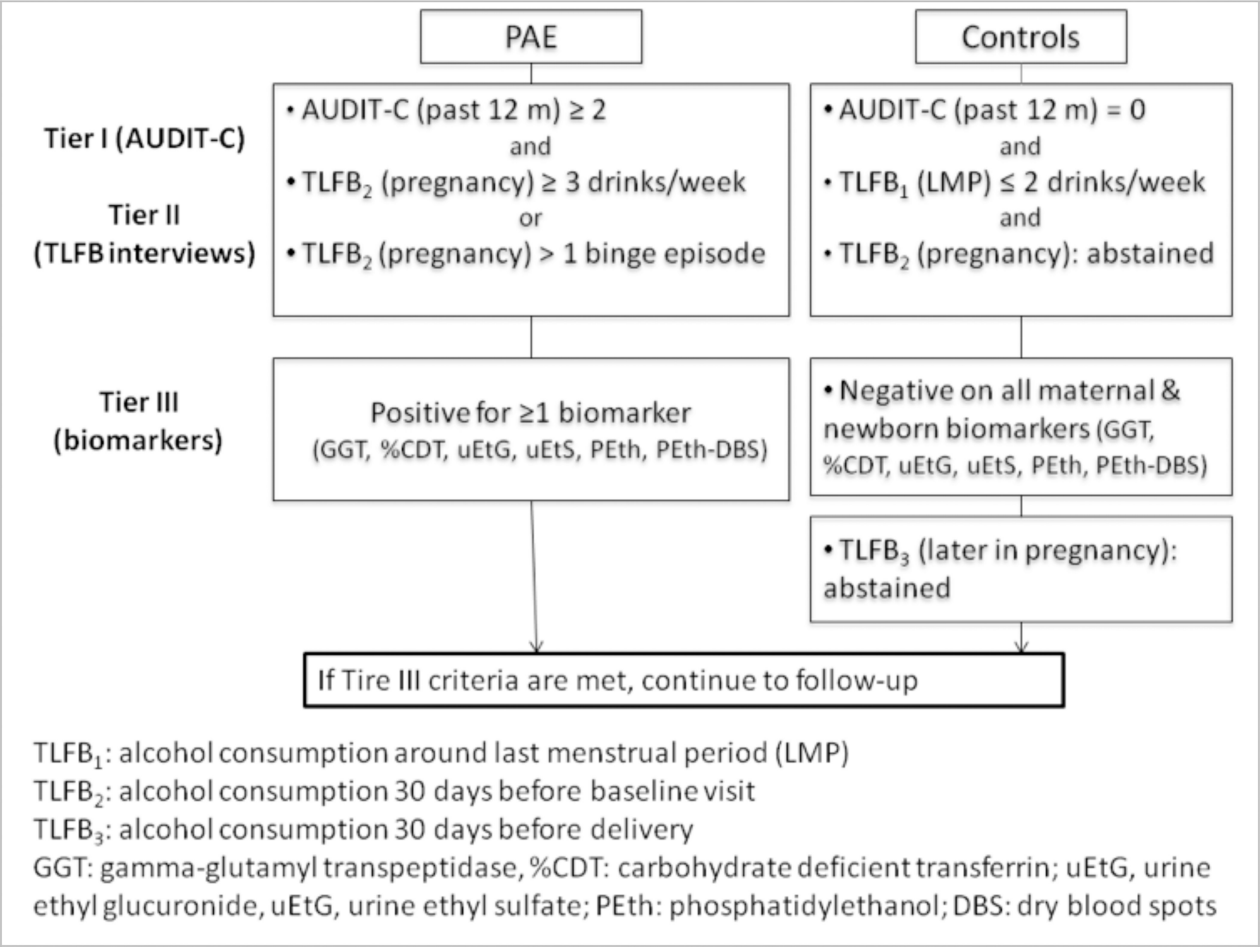
Screening and allocation into prenatal alcohol exposure and control groups

A three-tiered approached is used to assign patients to PAE and two control groups (Fig. 1). As an initial screener (Tier I), the AUDIT-C questionnaire is administered, consisting of three alcohol consumption items with a total score ranging from 0 to 12. Patients who score ≥2 are tentatively classified into the PAE group, while those with a score of zero are classified into one of the control groups and asked to provide informed consent. During the baseline interview, two 30-day timeline follow-back (TLFB) interviews are administered. The first TLFB calendar (TLFB_1_) covers 30 days around the LMP (two weeks before and two weeks after). The second TLFB calendar (TLFB_2_) covers 30 days prior to the baseline interview. Patients are also asked to report any binge drinking episodes (≥4 drinks/occasion) since LMP, and the last time they consumed any alcohol. To maintain eligibility as a control, a patient should report: 1) no more than two drinks per week in the periconceptional period on TLFB_1_; 2) no alcohol consumption since LMP; and 3) no drinking on the TLFB_2_ calendar (Tier II). Patients in the PAE group continue to be eligible if they report either at least one binge drinking episode or an average consumption of three drinks per week after LMP. For Tier III, exposure information is confirmed by a battery of ethanol biomarkers. In the mother, the panel includes gamma-glutamyltranspeptidase (GGT), carbohydrate-deficient transferrin (%dCDT), phosphatidylethanol (PEth), urine ethyl sulfate (uEtS), and urine ethyl glucurodine (uEtG) measured at both Visit 1 and Visit 2. In a newborn, an additional dry blood spot (DBS) card is collected at the same time of the routine newborn screen and analyzed for PEth (PEth-DBS). To remain eligible, controls should be negative for all biomarkers, while PAE group should have at least one positive biomarker to confirm exposure.

### Neurodevelopmental assessment

The Bayley Scales of Infant Development (third edition; BSID-III [24]) is the most widely used research tool to assess infant development. Overall standard scores in the composite areas of cognition, language, and motor skills are obtained.

Additional information is provided regarding fine versus gross motor skills, and receptive versus expressive language ability. This test is administered at both six and 20 months of age. A measure of object permanence is obtained from three items of the BSID-III cognitive scale. It has been found to be a culture-free way of measuring early working memory [25]. The ‘Snack Delay Self-Control’ task [26], in which the child is instructed not to touch a snack hidden under a cup, is administered to assess inhibition. The latency to touch the snack is scored with a maximum of 150 seconds. The A-not-B Task (adapted from Diamond [27]) is used to measure early working memory and involves hiding a toy under one of two cups placed in front of the child.

The still-face (SF) paradigm [28] is a measure of emotional regulation, self-regulation and stress reactivity. The SF design relies on an A-B-A model, in which A is normal play interaction, B is the SF episode, and the second A is a reunion/play episode. In this modified version, a second SF and third reunion/play are added (A-B-A-B-A model). SF episodes are coded for both infant affect and maternal responsiveness. Infant affect is coded into categories based on a scale adapted from the Infant Regulatory Scoring System (E.Z. Tronick and M.K. Weinberg, (1990). The Infant Regulatory Scoring System. Children’s Hospital and Harvard Medical School. Unpublished observations). Each second receives one (independent) affect score ranging from −3 (crying) to +3 (laughing), with 0 being neutral. Similar scales have been used in numerous studies to code infant affect [29, 30].

The mother’s interactive style during episodes 1, 3 and 5 (the episodes prior to the two SF episodes where she is allowed to interact) will be analyzed according to the coding system developed by Haley and Stansbury [31]. The coding of maternal sensitivity consists of an ordinal scale of variables including: 1) watching, 2) attention seeking, and 3) contingent responding. To evaluate inter-rater reliability, 10% of episodes are randomly selected, independently re-coded, and reliability is assessed based on agreement between coders for each second. Reliability coefficients averaged 0.88 for infant affect and parent responsiveness in prior studies [29, 32].

A ten-minute video of free play will provide us with the ability to look at mother-child interactions and child early play skills. Using Landry’s Scaffolding scale [33], (S.L. Landry, (2000). Mother-child coding manual for maternal targeted behaviors, child social responding, child social initiating. Unpublished observations), the mother’s verbalizations during the play interaction are coded to indicate whether they are trying to increase the child’s play by using scaffolding concepts (i.e., cause and effect, toy function, features of the toy). The Caregiver-Child Affective Responsiveness and Engagement Scale (C.S. Tamis-LeMonda, P. Ahuja, et al. (2011). Caregiver-child affect responsiveness and engagement scales (C-CARES). Unpublished observations) is a measure of maternal and child language, sensitivity, affect and dyadic interactions. The combination of these two coding schemas will provide information on effectiveness of the mother-child interaction, in addition to a child’s overall skills during the free play episode.

In addition, questionnaires are added that measure: children’s temperament (Infant Behavior Questionnaire-Revised (IBQ-R [34]) and Early Toddler Behavior Questionnaire (ECBQ [35]), sensory sensitivity (Infant/Toddler Sensory Profile, Parental Stress (PSI [36]) and maternal depression (Beck Depression Index; BDI-II [37]). These questionnaires will be compared to the other self-regulation assessments. Socio-economic status is assessed using the Barratt Simplified Measure of Social Status and provides estimate scores for education, occupation and overall SES [38].

### Assessment of sensory and motor development through MEG/EEG measurements

We obtain neurophysiological measures of auditory, somatosensory and motor development through simultaneous MEG/EEG measurements. Infants at six and 20 months of age are assessed with the Neuromag 306-channel MEG system and the Electrical Geodesics, Inc. 124-channel Hydrocell MEG-compatible EEG system. During data acquisition children are presented with simple auditory (800 Hz tone) and somatosensory (tactile stimulus to the right and left index finger) stimuli. The infant also participates in a *mu* rhythm suppression task, described in part in Berchicci *et al*. (2011) [39]. These tasks allow us to assess auditory and somatosensory peak amplitudes and latencies to determine if delays in sensory processing, as reported previously [22], indicate PAE based on biomarker and self-report measures. Furthermore, the *mu* rhythm suppression task allows us to assess development of the sensorimotor system and the neurophysiological markers of the infant’s ability to imitate simple adult motor skills relative to prenatal alcohol measures. The details of the MEG/EEG protocol are beyond the scope of the current report.

## Results

**Figure 2:**
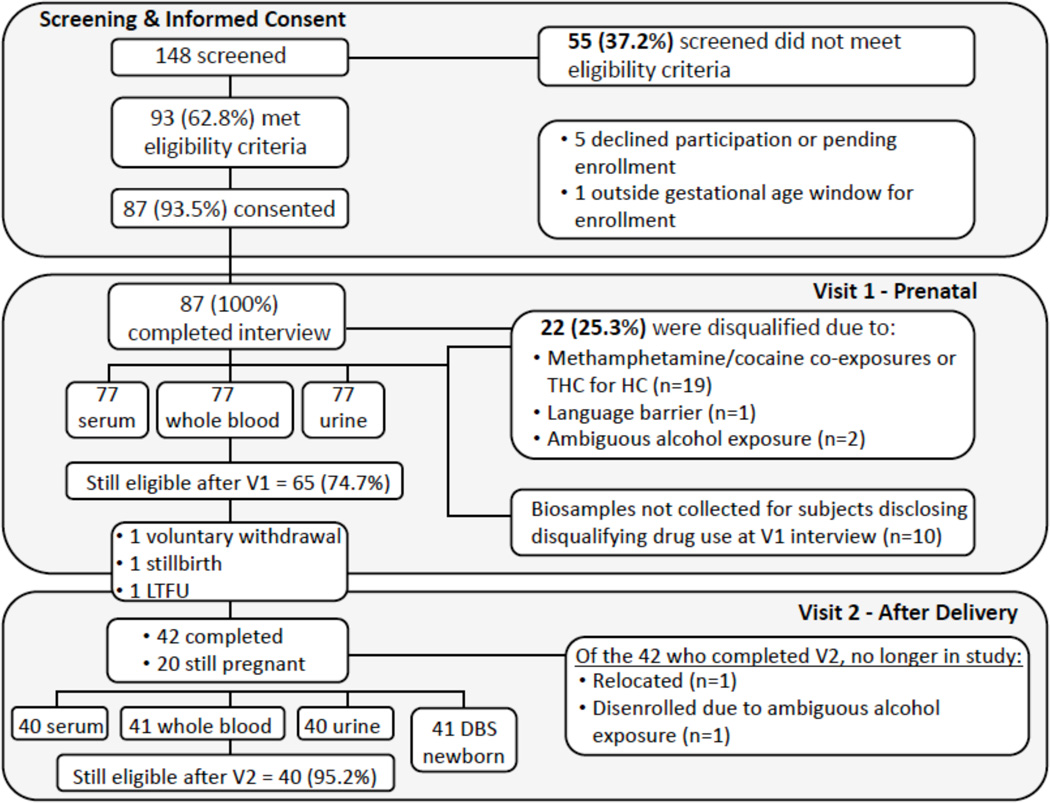
Recruitment and tracking of study participants

The recruitment and tracking of study participants in Year 1 are presented in Fig. 2. Out of 148 patients who were administered the screening interview, 62.8% met the eligibility criteria, and 93.5% of eligible patients went on to enroll in the study and participate in the baseline interview. After the baseline interview, a review of medical records and the Visit 1 urine drug screen, 74.7% of patients remained eligible; the remaining 25.3% of patients were disqualified and disenrolled from the study. The major disqualifier in our study population, given that the UNM Milagro clinic serves pregnant women with a history of substance abuse, is concurrent use of methamphetamine or cocaine (25.3%). In addition, two patients were disqualified after the baseline interview because they no longer met the eligibility criteria with respect to alcohol use for a specific study group. The biospecimen collection rate was 100% at Visit 1, and 97.6% for those who completed Visit 2 (one complete set of samples was not collected due to delivery outside UNM Hospital).

The demographic characteristics of 87 participants who completed the Visit 1 assessment are presented in Table 1. The mean maternal age at recruitment was 27.7±5.9 years.

Most patients were recruited during the middle of the second trimester (22.5±6.7 gestational weeks), and the mean gestational age at recruitment was similar among the three study groups (*p*=0.63).

There were no differences between the study groups with respect to maternal age, race, ethnicity, or employment status (all *p*-values >0.05).

Some differences were observed in marital status and education level (*p*<0.01), with a higher proportion of single women among alcohol-exposed and OMT controls, and a higher proportion of women with at least a college education among healthy controls. In addition, some differences were observed among groups with respect to the health insurance status (*p*<0.01), with much higher prevalence of employer-based insurance among healthy controls compared to other two groups.

## Discussion

### Assessment of alcohol exposure

In this study we have chosen to limit the alcohol-exposed group to participants with at least one confirmed alcohol biomarker (in either maternal or newborn specimens), and to exclude children whose mothers admit to drinking during pregnancy but test negative for biomarkers. We acknowledge that this approach might exclude women who discontinued or substantially reduced drinking after pregnancy recognition. However, due to the limited size of this initial study cohort, we are intentionally limiting the PAE group to subjects who are ‘positive’ on both self-report and ethanol biomarkers. This is necessitated by the importance to first identify indices of atypical brain development in young children with documented PAE, which has not previously been done in a prospective longitudinal study in the US. We envision expanding the study to include children with PAE early in gestation, and those with a moderate-to-light level of exposure, whose mothers discontinued or substantially reduced alcohol use upon pregnancy recognition.

We also recognize that other methods to assess PAE are available, in addition to the measures employed in this study. We chose AUDIT-C as an initial screening tool to minimize patient burden prior to the consent.

AUDIT-C has demonstrated approximately equal accuracy to the full ten-item AUDIT questionnaire and has been shown to be an effective screening tool to identify hazardous drinking in women (reviewed by Reinert, 2007 [40]). In pregnant women, AUDIT-C demonstrated an area under the curve (AUC) of 0.97 for correct identification of risk drinking in the past year [41].

After this initial brief screening and consent, potentially eligible patients undergo rigorous evaluation of alcohol consumption pattern by administering three 30-day TLFB interviews - a current ‘gold standard’ for assessment of PAE. It should be noted that controls in our study are not lifelong abstainers, rather they included light drinkers (≤2 drinks/week) during the periconceptional period who abstained from alcohol during pregnancy. We felt that such a control group would better represent a typical low-risk population of pregnant women than life-long abstainers.

The battery of maternal biomarkers has been identified for the following reasons: a) %CDT is an established biomarker and the only Food and Drug Administration (FDA)-approved biomarker for assessing hazardous alcohol use; b) GGT, while not specific, might be more sensitive than CDT in women [42] and has the widest detection window; c) PEth has emerged as a novel and highly sensitive and specific biomarker [43, 44]) uEtG and uEtS were chosen as sensitive biomarkers for identification of recent alcohol consumption [45]. These biomarkers have different detection windows (i.e., 1–2 months for GGT; 4–5 weeks for %CDT, around three weeks for PEth, and <4 days for uEtG/uEtS); thus, can capture different patterns of alcohol use in the study population [46]. In addition, PEth-DBS was chosen as a biomarker of choice in the newborn given recent reports of its high sensitivity and specificity [43] and limitations of meconium biomarkers, such as high false positive rate among meconium fatty acid ethyl esters [47].

It should also be acknowledged that our PAE group includes patients on OMT (methadone or buprenorphine). Prior studies in the FASD field acknowledge that the use of other substances, e.g., tobacco [48], cocaine, marijuana, and opiates [49–51], is very prevalent among alcohol-using pregnant women. Recent studies reported that as many as 23% of pregnant women might report substance use [52], and neonates with heavy *in utero* ethanol exposure are 2–3 times more likely to be exposed to opiates and amphetamines compared to unexposed children [53]. Thus, the problem of concurrent use of other substances cannot be ignored in the FASD research field. While prenatal opiate exposure might affect neonatal fetal growth and is often associated with neonatal abstinence syndrome during the first month of life, its effect on longer-term behavioral outcomes is expected to be lower compared to alcohol or other classes of illicit drugs (reviewed by Davies, 2005 [54]). Thus, to minimize variability in co-exposures with different substances, women in the PAE group are co-exposed to opioids, while co-exposures to amphetamines and cocaine (assessed by repeated interviews and urine drug screens) are study disqualifiers. In addition, the OMT control group will allow us to account for the effects of prenatal opioid exposure alone, relative to the control group.

### Selection of neurocognitive tests

The BSID-III provides a reliable method to determine whether children are following a typical developmental course across the first 20 months of age. The BSID-III is the most common research tool for establishing development in young infants and allows us to compare results to the current literature. As mentioned, the still-face paradigm was chosen to assess emotion regulation at six months of age. Haley *et al*. have previously found that children with PAE at 5–7 months of age had increased stress reactivity [55]. Children diagnosed with FASD also have increased risk for difficulty with attention and executive function [56]. Early difficulty with self-regulation could be related to later problems with social behavior found in FASD groups [57].

Play in early years of life is both a measure of mother-infant interaction and early development, thereby motivating the use of the play paradigm at the 20-month visit. Parents’ use of verbal scaffolding in play has been associated with development of verbal IQ in preterm children both at 18 months and at three years of age [29, 58, 59]. Scaffolding, first described by Vygotsky, is a way of increasing a child’s abilities through the demonstration of higher skills by a caregiver or teacher.

Symbolic play has been found to be an early indicator of developmental delays in young children with FASD [60]. In a study by Molteno *et al*. (2010), 13-month old toddlers who were able to use higher levels of elicited symbolic play demonstrated better early memory skills at five years of age. Elicited play was also found to be a moderate predictor of verbal IQ at 7.5 years [61].

The role of mother-child interaction during play was also associated with early working memory and cognition in 18-month old preterm children using the Caregiver Child Affect, Responsiveness and Engagement Scale (C-CARES [62]). It is important to note that FASD cannot be reliably diagnosed at the age assessed in this current study. That is, the cognitive functions associated with the neurobehavioral profile associated with FASD classification have not yet been fully developed to allow for assessment of normal or abnormal performance at 20 months of age.

### Potential confounders and other limitations

As with all human studies we acknowledge a number of potential confounders that may increase the variability in brain maturation within and across groups. These include variability in maternal and infant nutrition, infant rearing environment, maternal stress, postpartum depression, infant-maternal bonding, premature birth, and genetic factors. Within the current design we collect a number of measures to allow us to capture indicators of many of these factors to allow for comparisons both within and across groups.

Some differences in the demographic characteristics observed among the study groups emphasize the importance of having a second control group: opioid-dependent women who abstain from alcohol use in pregnancy. As demonstrated in Table 2, these women have comparable or even lower socio-economic characteristics as compared to alcohol-exposed patients. We believe that this group will allow us to largely control for both pre- and postnatal risk factors associated with PAE.

Given the well-known concern in prenatal alcohol studies of caloric replacement in heavy drinkers, a validated Block food frequency questionnaire [63] is administered at Visit 2 to capture participants’ caloric and micronutrient intake, as well as controlling for SES across groups. We track maternal stress and postpartum depression measures through questionnaires completed at Visit 2, i.e., the Perceived Stress Scale [64, 65], and at Visits 3 and 4, i.e., Beck Depression Inventory, and the Parenting Stress Index. Furthermore, infant-maternal interaction is captured through performing the still-face paradigm in six-month old children and free play at 20 months.

We have not excluded preterm infants (<37 weeks of gestation) from the current study. While prematurity is a risk factor for neurodevelopmental delays, infants with prenatal alcohol and drug exposure are also at higher risk for preterm birth, thereby requiring that we retain these children for the study. The infants must not have a complicated course, e.g. they are excluded if their preterm birth necessitates care in the neonatal intensive care unit (NICU). This exclusion limits the number of confounding factors that may impact brain development due to prematurity. Furthermore, preterm infants are included for all groups and prevalence of prematurity will be closely monitored in each of the three study groups.

Finally, we know that genetic makeup alters the influence of PAE on fetal development. Individuals with the ADH1B*3 allele are known to metabolize alcohol more quickly than those without the polymorphism [66]. This fast metabolism eliminates the toxicant from the body more quickly and thus plays a protective role against PAE [67, 68]. However, this polymorphism is primarily found in individuals of African descent [69]. The population in Albuquerque, New Mexico is both ethnically and racially diverse with ~50% non-Hispanic White, 40% with Hispanic ethnicity and 10% Native Americans. Yet, African Americans make up only a small portion of the population of NM (1–2%), thereby limiting our ability to stratify genetic profiles based on already identified factors and reducing the likelihood that we will identify individuals with this protective genetic profile.

### Ethical considerations

This study was approved by the Human Research Review Committee at the University of New Mexico Health Sciences Center. The study is initially presented to the mothers during a routine prenatal visit. All study participants who report alcohol use are provided with a brochure from the MotherToBaby Counseling service [70] at the end of the baseline interview. Since alcohol-using patients are recruited from an established UNM substance abuse program for pregnant mothers, they are already closely monitored by prenatal care specialists and psychiatrists, and are provided with counseling to reduce the risk to the child. Mothers and their infants benefit from the study by receiving a written summary from the Certified Diagnostician (author JL) with respect to the child’s development at six and 20 months. Children with developmental delays are referred to the free early intervention programs available in New Mexico. To provide legal protection for the study participants a Certificate of Confidentiality was obtained from the National Institutes of Health (NIH). This is intended to protect individuals from prosecution based on identification of illicit drug use that is explicitly tested as a part of this study. Finally, it should be noted that the Certificate of Confidentiality does not protect parents from mandatory reporting of suspected child abuse according to New Mexico law.

### Unique strengths

A unique strength of the current study is the use of MEG and EEG to assess functional brain development in very young children. MEG is a noninvasive functional neuroimaging technique that provides excellent temporal resolution of brain dynamics and good spatial resolution. In comparison to functional magnetic resonance imaging (fMRI), MEG and EEG provide superior temporal resolution allowing one to assess temporal processing delays within cortical networks. Furthermore, functional measures of brain development using MRI are currently obtained in children of approximately five years and older due to the challenges associated with collecting awake functional data within the restricted MR environment. MEG and EEG provide a quiet data collection environment within which to collect functional data from awake infants while maintaining a welcoming environment for young children [71]. In comparison to EEG, MEG provides superior spatial resolution and is not sensitive to skull features, such as the skull fontanels, that add additional variability to EEG data when performing comparisons across subjects and performing longitudinal studies. Finally, this study will obtain measures of neurodevelopment assessed through standardized neurodevelopmental tests such as the BSID-III and tests of early working memory [25] that will in turn be correlated with neuroimaging measures of sensory response latency [22] and cortical development [39].

## Figures and Tables

**Table 1:** Summary of neuroimaging and neurodevelopmental assessments

Cognitive– behavioral domains	6 months	20 months	Potential Confounders
Sensory processing	MEG sensory*	MEG sensory*	Socio- demographic characteristics
	Sensory Profile	Sensory Profile	Parenting
Cognitive and early working memory	BSID-III	BSID –III A-not-B task*	Stress Index- short form Beck’s
Self- regulation	Still-face paradigm*	C-CARES* Snack Delay*	Depression Index
	IBQ-R	ECBQ	Maternal language
Motor and cortical connectivity	MEG (mu rhythm)*	MEG (mu rhythm)*	(C-CARES)
	BSID-III Motor Scale	BSID- III Motor Scale	

* Assessments marked by asterisk indicate experimental paradigms

**Table 2:** Demographic characteristics of eligible participants (n=87)

	Healthy Control (n=21)	OMT Control (n=37)	Alcohol +/− OMT (n=29)	*p*-value
	Mean±SD	Mean±SD	Mean±SD	
Maternal age in years, mean	26.8±6.3	27.7±5.9	28.5±5.8	0.38^1^
Gestational age at recruitment	24.0±7.0	22.3±6.6	21.7±6.8	0.63^1^
	N (%)	N (%)	N (%)	
Marital status:				<0.01^2^
Single, never married	6 (28.6%)	23 (62.2%)	14 (48.3%)	
Married, living with spouse	11 (52.4%)	4 (10.8%)	11 (37.9%)	
Not married, living with partner	2 (9.5%)	8 (21.6%)	4 (13.8%)	
Divorced/Separated	2 (9.5%)	2 (5.4%)	0	
Hispanic, Latino, Spanish	11 (52.4%)	29 (78.4%)	21 (72.4%)	0.11^2^
Race:				0.60^2^
White	15 (71.4%)	30 (81.1%)	19 (65.5%)	
Black or African American	0	0	2 (6.9%)	
American Indian	1 (4.8%)	2 (5.4%)	2 (6.9%)	
Multi-racial/Other/Prefer not to answer	5 (23.8%)	5 (13.5%)	6 (20.7%)	
Highest level of school completed:				<0.01^2^
High school graduate or less	6 (28.6%)	28 (75.7%)	14 (48.3%)	
Some college/vocational school	6 (28.6%)	9 (24.3%)	9 (31.0%)	
College degree or higher	9 (42.9%)	0	6 (20.7%)	
Currently employed:	10 (47.6%)	8 (21.6%)	9 (31.0%)	0.13^2^
Health Insurance status:				
No insurance	1 (4.8%)	0	1 (3.4%)	<0.01^2^
Employer-based insurance	9 (42.9%)	2 (5.4%)	4 (13.8%)	
Medicaid/Other public	11 (52.4%)	35 (94.6%)	24 (82.8%)	

1 ANOVA test for equality of means

2 Fisher’s exact test

OMT, opioid maintenance therapy
